# Sociodemographic determinants of sexuality among transmasculine and transfeminine individuals: A cross‐sectional study in São Paulo, Brazil

**DOI:** 10.1002/ijgo.71081

**Published:** 2026-05-16

**Authors:** Augusto Rimoli Esteves, Gabriel de Castro Pereira Bittar, Bruna Marques Lopes, Sônia Maria Rolim Rosa Lima

**Affiliations:** ^1^ Faculty of Medical Sciences Santa Casa de São Paulo São Paulo Brazil

**Keywords:** gender identity, sex work, sexual partners, sexuality, sociodemographic factors, transgender health

## Abstract

**Objective:**

This study assessed the associations between gender identity (transmasculine vs transfeminine) and sexuality‐related variables, including marital status, sexual partners, sex work, and age at first intercourse.

**Methods:**

We conducted a cross‐sectional retrospective study at a specialized transgender clinic in São Paulo, Brazil, analyzing medical records from March 2020 to August 2023. Data were routinely collected during clinical consultations through standardized questions for individualized care. Group assignment was based on self‐identified gender identity documented in medical records. Chi‐square and *t*‐tests were applied (*P* < 0.05).

**Results:**

We evaluated 74 individuals (trans men = 49, trans women = 25). Trans women were more frequently single (76% vs 44.9%, *P* = 0.014). Most reported one to five partners, but trans women more frequently reported no partners (16.7%) or >15 partners (11.1%) (*P* = 0.043). Sex work was more common among trans women (21.7% vs 6.3%, *P* = 0.049). No significant difference in age at first intercourse was observed (*P* = 0.298).

**Conclusion:**

Gender identity significantly influences relationship status, sexual behavior, and sex work engagement among transgender individuals. Trans women experienced greater sexual vulnerability, reflecting social exclusion and structural transphobia. These findings underscore the need for inclusive sexual health strategies and policies reducing structural inequalities.

## 1 INTRODUCTION

Sexuality is central to human life, deeply influenced by social, economic, and cultural contexts. For transgender individuals, sexual and affective experiences are shaped by gender identity, stigma, and barriers to social inclusion.^1^ The transgender population faces disproportionate violence, limited healthcare access, and labor market exclusion, directly impacting sexual behavior and health outcomes.^2^, ^3^

Marital status, number of sexual partners, sex work engagement, and age at first sexual intercourse are key indicators identifying vulnerability patterns and informing public health strategies.^4^ Age at first sexual intercourse is important for assessing sexual education and reproductive planning.^5^

A 2021 survey showed that 90% of Brazil's trans population resorts to prostitution as primary income.^6^ Silva et al. observed that 71.7% of transgender individuals had never married.^7^ A São Paulo State University study revealed that 64.7% of transgender participants were not in relationships.^8^

This study compares transmasculine and transfeminine individuals regarding sexuality‐related sociodemographic factors, based on data from a specialized transgender health clinic in São Paulo, Brazil, to understand how gender identity intersects with these variables and their implications for Brazilian public health policies.

## 2 MATERIALS AND METHODS

### 2.1 Study design, setting, and period

This cross‐sectional, retrospective study was conducted at the Barra Funda School Health Center, a partner unit of the Primary Care Department of the Santa Casa de São Paulo School of Medical Sciences, located in São Paulo, Brazil. This clinic provides specialized care for transgender individuals, including hormone therapy, psychological support, and surgical referrals. Data were collected retrospectively from medical records of patients attending the transgender outpatient clinic between March 1, 2020, and August 30, 2023.

### 2.2 Participants and data source

Inclusion criteria were patients who self‐identified as transmasculine (trans men) or transfeminine (trans women), were aged ≥18 years, and had sociodemographic forms with at least one variable completed. Exclusion criteria were patients with incomplete medical records lacking documentation of gender identity and those who self‐identified as nonbinary or as other gender identities outside the transmasculine/transfeminine categories.

All data were extracted from medical records. During routine clinical consultations, healthcare providers systematically collected sociodemographic information through standardized questions to provide individualized, patient‐centered care.

A total of 92 patients attended the clinic during the study period, and the sample size was determined by including all patients who met the inclusion criteria and were followed at the studied health service. Of these, 74 participants met the inclusion criteria and had sociodemographic and gender identity data recorded (49 trans men, 25 trans women). Eighteen participants were excluded: seven for self‐identifying as nonbinary, nine for incomplete forms or missing data, and two for being younger than 18 years (Figure 1). Variation in sample sizes among Tables 1, 2, and 3 reflects the real‐world clinical context in which not all patients responded to all questions, which is typical of retrospective studies using routine clinical data.

**FIGURE 1:**
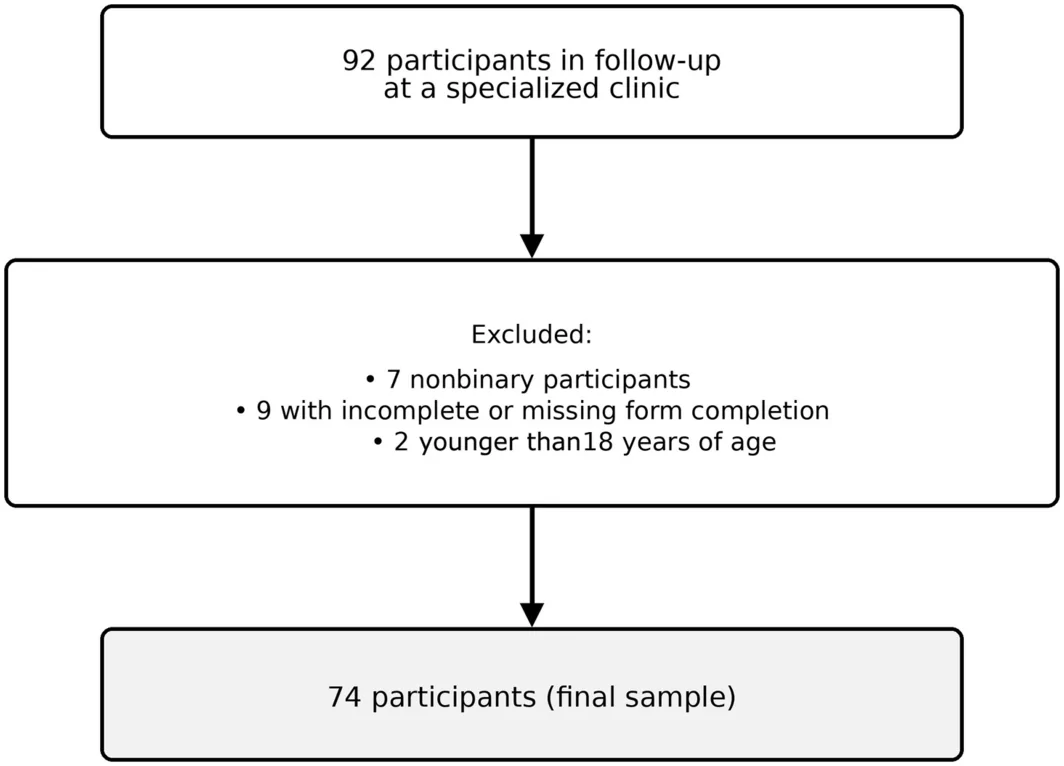
Flowchart for patient selection after applying inclusion and exclusion criteria.

**TABLE 1:** Comparative study between the group of trans women and the group of trans men regarding marital status (*n* = 74). Barra Funda School Health Center—FCMSCSP.

Marital status	Gender				Total	
	Trans women		Trans men			
Married or cohabiting	4	16.0%	13	26.5%	17	23.0%
Dating	1	4.0%	14	28.6%	15	20.3%
Single	19	76.0%	22	44.9%	41	55.4%
Divorced	1	4.0%	0	0.0%	1	1.0%
Total	25	100.0%	50	100.0%	74	100.0%

*Note*: χ^2^ *P* = 0.014.

**TABLE 2:** Comparative study between the group of trans women and the group of trans men regarding the number of sexual partners in the past 12 months (*n* = 64). Barra Funda School Health Center—FCMSCSP.

Number of sexual partners (in the last 12 months)	Gender				Total	
	Trans women		Trans men			
None	3	16.7%	0	0.0%	3	4.7%
1–5	11	61.1%	31	67.4%	42	65.6%
6–10	1	5.6%	6	13.0%	7	10.9%
11–15	0	0.0%	3	6.5%	3	4.7%
>15	2	11.1%	1	2.2%	3	4.7%
Don't know	1	5.6%	5	10.9%	6	9.4%
Total	18	100.0%	46	100.0%	64	100.0%

*Note*: χ^2^ *P* = 0.043.

**TABLE 3:** Comparative study of the group of trans women and the group of trans men regarding having had sex in exchange for money (*n* = 72). Barra Funda School Health Center—FCMSCSP.

Engagement in sex work	Gender				Total	
	Trans women		Trans men			
No	18	78.3%	45	93.8%	63	88.7%
Yes	5	21.7%	3	6.3%	8	11.3%
Total	23	100.0%	48	100.0%	72	100.0%

### 2.3 Gender identity definition and group assignment

Gender identity was self‐reported during clinical consultations and documented in medical records. Healthcare providers asked: “What is your current gender identity?” Responses were recorded in standardized fields.

Transmasculine individuals (trans men) were defined as patients assigned female at birth who currently identify as male or transmasculine. Transfeminine individuals (trans women) were defined as patients assigned male at birth who currently identify as female or transfeminine. Patients who self‐identified as nonbinary were excluded because the study aimed to compare binary transgender identities. Group assignment was based exclusively on self‐identified current gender identity as documented in medical records, not on medical transition status, hormone use, or surgical history.

### 2.4 Routine data collection protocol

Sociodemographic data were collected as part of routine clinical practice through a dedicated form during initial consultations. Healthcare providers asked standardized questions covering demographic information, social and family support, marital status and relationships, sexual health history, and economic situation and income sources including sex work, housing stability, and healthcare access. Questions were asked sensitively by trained professionals. Patients were informed that providing information was voluntary and would help provide better care. Responses were documented directly in medical records.

The systematic collection of sociodemographic information serves multiple clinical purposes: individualized care planning by understanding each patient's social context enables healthcare providers to tailor interventions to individual needs; risk assessment by identifying vulnerabilities allows for targeted screening and prevention strategies; and continuity of care by documenting information ensures all team members have access to relevant information for coordinated care.

### 2.5 Variables and outcomes

This study focused on four sociodemographic variables related to sexuality, selected based on their relevance to transgender sexual health^1^, ^4^, ^6^, ^9^ and availability in medical records: marital status (categorized as single, dating, or married/cohabiting), number of sexual partners in the past 12 months (categorized as 0, 1–5, 6–10, 11–15, or >15 partners), engagement in sex work (binary variable: yes/no), and age at first sexual intercourse in years.

### 2.6 Missing data

Missing data were handled using complete‐case analysis, in which patients with missing documentation for specific variables were excluded only from analyses involving those variables. The variation in sample sizes reflects routine clinical data collection, as not all patients responded to every question. Contributing factors included patient comfort, clinical priorities, and variability in documentation practices. Accordingly, sample sizes varied across analyses: Table 1 (marital status) *n* = 74 (0 missing), Table 2 (sexual partners) *n* = 64 (10 missing), and Table 3 (sex work) *n* = 72 (2 missing). No imputation methods were used.

### 2.7 Confidentiality and data protection

To protect patient confidentiality, each patient was assigned a unique anonymized identification code. Personal identifiers were not extracted. Data extraction was performed by authorized research team members. All extracted data were stored in password‐protected files complying with Brazilian General Data Protection Law.

### 2.8 Statistical analysis

Descriptive statistics were calculated for all variables. Continuous variables are expressed as mean ± standard deviation (SD) and compared using independent‐samples *t*‐tests. Categorical variables are expressed as frequencies and percentages and compared using Chi‐squared tests or Fisher exact test when expected cell counts were <5. A significance level of *P* < 0.05 was adopted. All analyses were performed using SPSS version 13.0 (IBM).

### 2.9 Ethical considerations

This study was conducted in accordance with the Declaration of Helsinki and approved by the research ethics committee of the Faculty of Medical Sciences of Santa Casa de São Paulo (CAAE: 69239723.2.0000.5479; approval number: 6.060.670). As this was a retrospective study using de‐identified data from routine clinical care, the Research Ethics Committee granted a waiver of informed consent.

## 3 RESULTS

A total of 74 patients were included: 49 trans men (66.2%) and 25 trans women (33.8%). The mean age of the population was 28.4 ± 6.7 years (trans men: 27.8 ± 6.3 years; trans women: 29.6 ± 7.4 years [*P* = 0.276]). There was a statistically significant association between gender identity and marital status (*χ* ^2^ = 8.56, df = 2; *P* = 0.014). Among trans women, 76.0% (*n* = 19) were single, 16.0% (*n* = 4) were married or cohabiting, and 8.0% (*n* = 2) were dating. Among trans men, 44.9% (*n* = 22) were single, 28.6% (*n* = 14) were dating, and 26.5% (*n* = 13) were married or cohabiting (Table 1).

The number of sexual partners showed a statistically significant association with gender identity (*χ* ^2^ = 9.84, df = 4; *P* = 0.043). Most participants reported one to five partners (trans women: 61.1%, *n* = 11; trans men: 67.4%, *n* = 31). However, trans women more frequently reported no sexual partners (16.7%, *n* = 3) compared with trans men (4.3%, *n* = 2). Additionally, trans women more frequently reported >15 partners (11.1%, *n* = 2) compared with trans men (2.2%, *n* = 1) (Table 2).

Engagement in sex work showed a statistically significant association with gender identity (*χ* ^2^ = 3.89, df = 1; *P* = 0.049). Trans women reported sex work engagement in 21.7% (*n* = 5) of cases, while trans men reported this in 6.3% (*n* = 3) (Table 3).

There was no significant difference in mean age at first sexual intercourse between groups (trans women: 16.2 ± 3.1 years; trans men 15.6 ± 2.8 years [*t* = 1.04, df = 72; *P* = 0.298]).

## 4 DISCUSSION

This study demonstrates that gender identity is associated with important aspects of sexual behavior and affective relationships among transgender individuals in São Paulo, Brazil. Trans women were more frequently single, had more polarized sexual activity patterns, and were significantly more engaged in sex work compared with trans men.

Higher sex work rates among transfeminine individuals (21.7% vs 6.3%) may reflect systemic employment barriers.^9^ In Brazil, transgender women face severe labor market discrimination, with unemployment rates as high as 90% in some regions.^6^ Although sex work is not inherently negative, in this context it often reflects a lack of alternatives. Structural interventions must expand labor opportunities and protect the rights of those engaging in sex work.

Greater proportions of stable relationships among trans men (55.1% in dating or married/cohabiting relationships vs 24.0% among trans women) may be associated with higher social acceptance. Some studies suggest that trans men may be more readily perceived as cisgender in social interactions, which may reduce relational stigma.^10^ Trans women more frequently reported either no sexual partners (16.7%) or very high numbers (>15: 11.1%) compared with trans men, suggesting polarization reflecting both social isolation and engagement in sex work or hypersexualized contexts.

No significant difference in age at first sexual intercourse was observed between groups (trans women: 16.2 years; trans men: 15.6 years; *P* = 0.298), suggesting that sexual initiation may occur at similar ages regardless of gender identity. Both groups reported ages consistent with the general Brazilian population,^5^ suggesting that transgender identity per se may not influence the timing of sexual debut.

### 4.1 Implications for public health

These findings have important public health implications. Trans women require specific interventions addressing social exclusion, economic vulnerability, and sexual health risks. Healthcare providers should be trained to address diverse sexual health needs without stigma. Policies reducing employment discrimination and expanding economic opportunities for transgender individuals, particularly trans women, are urgently needed. Mental health and social support services should address challenges in forming and maintaining romantic relationships. The systematic collection of sociodemographic information during routine clinical care can serve as a model for other transgender health services.

### 4.2 Limitations

This study has several limitations. The relatively small sample size (*n* = 74) from a single specialized clinic limits statistical power and generalizability to only the specific clinic population, not the broader Brazilian transgender population. The cross‐sectional design does not allow causal inferences. As a retrospective study using routine clinical data, we were limited to information documented in medical records and could not verify accuracy or obtain additional data not routinely collected. The study included only patients who attended the clinic; transgender individuals who do not access healthcare services are not represented. Although the missing data proportion was small (<15% for any variable), we cannot rule out the possibility that data were missing not at random, potentially introducing bias.

All data were based on patient self‐report and may be subject to recall or social desirability bias, particularly for sensitive topics. The exclusion of nonbinary patients limits the scope of our findings and does not reflect full diversity of gender identities. The analysis focused on only four sexuality‐related variables routinely documented; more comprehensive analysis would provide a richer understanding.

### 4.3 Strengths and contributions

Despite limitations, this work provides important contributions. To our knowledge, this is the first Brazilian study to systematically describe and compare sexuality‐related sociodemographic variables between transmasculine and transfeminine individuals. These findings highlight specific vulnerabilities, inform development of inclusive public health strategies, and may serve as a foundation for future research, thereby strengthening the evidence base on transgender health in Brazil.

## 5 CONCLUSION

This study demonstrates that gender identity significantly influences relationship status, sexual behavior, and sex work engagement among transgender individuals attending a specialized clinic in São Paulo, Brazil. Trans women demonstrated greater sexual vulnerability, reflecting social exclusion and structural transphobia. Future multicenter studies with larger samples and prospective data collection are needed to confirm and extend these findings.

## AUTHOR CONTRIBUTIONS

All authors contributed to the initial design of the work and question formation. **Augusto Rimoli Esteves:** Conceptualization; Data collection during clinical consultations; Formal analysis; Investigation; Methodology; Project administration; Writing – original draft; Writing – review & editing. **Gabriel de Castro Pereira Bittar:** Data collection during clinical consultations; Data extraction from medical records; Investigation; Writing – review & editing. **Bruna Marques Lopes:** Data curation; Investigation; Writing – review & editing. **Sonia Maria Rolim Rosa Lima:** Conceptualization; Methodology; Project administration; Supervision; Validation; Writing – review & editing.

## FUNDING INFORMATION

The authors received no specific funding for this work.

## CONFLICT OF INTEREST STATEMENT

The authors declare no conflicts of interest.

## ETHICS STATEMENT

This study was conducted in accordance with the guidelines of the Declaration of Helsinki and approved by the research ethics committee of the Faculty of Medical Sciences of the Santa Casa of São Paulo (CAAE: 69239723.2.0000.5479), having been assigned registration number 6.060.670 in the national Plataforma Brasil. A waiver of informed consent was granted for this retrospective study using deidentified data from routine clinical care.

## Supporting information


Data S1.

Data S2.

## Data Availability

The data that support the findings of this study are available from the corresponding author upon reasonable request. Due to ethical restrictions and privacy concerns related to the use of patient medical records, raw data cannot be shared publicly but can be made available to qualified researchers under appropriate data sharing agreements and with approval from the institutional ethics committee.

## References

[1] World Health Organization (WHO) . Sexual Health, Human Rights and the Law . 2015. Accessed October 5, 2022. https://iris.who.int/bitstream/handle/10665/175556/9789241564984_eng.pdf

[2] Grant JM , Mottet LA , Tanis J , Harrison J , Herman JL , Keisling M . Injustice at Every Turn: A Report of the National Transgender Discrimination Survey . 2011. Accessed October 5, 2022. https://www.thetaskforce.org/app/uploads/2019/07/ntds_full.pdf

[3] Reisner SL , Hughto JM , Dunham EE , et al. Legal protections in public accommodations settings: a critical public health issue for transgender and gender‐nonconforming people. Milbank Q. 2015;93(3):484‐515. doi:10.1111/1468-0009.12127 PMC4567851 26219197

[4] World Health Organization (WHO) . Developing Sexual Health Programmes: A Framework for Action . 2010. Accessed October 5, 2022. https://iris.who.int/bitstream/handle/10665/70501/WHO_RHR_HRP_10.22_eng.pdf

[5] Janis JA , Ahrens KA , Ziller EC . Female age at first sexual intercourse by rural–urban residence and birth cohort. Womens Health Issues. 2019;29(6):489‐498. doi:10.1016/j.whi.2019.07.004 31444037

[6] Benevides BG . Dossiê assassinatos e violências contra travestis e transexuais brasileiras em 2021. Associação Nacional de Transexuais e Travestis (ANTRA); 2022. Accessed October 5, 2022. https://antrabrasil.files.wordpress.com/2022/01/dossieantra2022‐web.pdf

[7] Silva DC , Rabelo‐da‐Ponte FD , Salati LR , Lobato MIR . Heterogeneity in gender dysphoria in a Brazilian sample awaiting gender‐affirming surgery: a data‐driven analysis. BMC Psychiatry. 2022;22(1):79. doi:10.1186/s12888-022-03727-3 PMC8808968 35105327

[8] Spizzirri G , Eufrásio R , Lima MCP , et al. Proportion of people identified as transgender and non‐binary gender in Brazil. Sci Rep. 2021;11:2240. doi:10.1038/s41598-021-81411-4 PMC7838397 33500432

[9] Poteat T , German D , Flynn C . The conflation of gender and sex: gaps and opportunities in HIV data among transgender women and MSM. Glob Public Health. 2016;11(7–8):835‐848. doi:10.1080/17441692.2015.1134615 PMC4957661 26785751

[10] Budge SL , Katz‐Wise SL , Tebbe EN , Howard KAS , Schneider CL , Rodriguez A . Transgender emotional and coping processes: facilitative and avoidant coping throughout gender transitioning. J Couns Psychol. 2013;60(4):601‐647. doi:10.1177/0011000011432753

