# Supplementary material for: Sociodemographic determinants of sexuality among transmasculine and transfeminine individuals: A cross‐sectional study in São Paulo, Brazil
Source: Int J Gynaecol Obstet. 2026 May 16;175(2):756–61. doi: 10.1002/ijgo.71081 (PMC13674826; doi:10.1002/ijgo.71081)
Supplement: Supplementary file 1 [file IJGO-175-756-s001.docx]

# Clinical Sociodemographic Form – Trans Health Clinic

## 1. Identification

Social name: ______________________________________________

Legal name (optional): _______________________________________

## 2. Gender Identity

☐ Trans man / Transmasculine

☐ Trans woman / Transfeminine

☐ Non-binary

☐ Other: ___________________________________

☐ Prefer not to answer

## 3. Race/Ethnicity

☐ White

☐ Black

☐ Brown / Mixed-race (Parda)

☐ Asian

☐ Indigenous

☐ Prefer not to answer

## 4. Education Level

☐ Incomplete elementary school

☐ Complete elementary school

☐ Incomplete high school

☐ Complete high school

☐ Incomplete higher education

☐ Complete higher education

☐ Postgraduate degree

☐ Prefer not to answer

## 5. Study Status

☐ Currently studying

☐ Not studying

## 6. Work Status

☐ Formal employment

☐ Informal employment

☐ Self-employed

☐ Unemployed

☐ Retired

☐ Student

☐ Other: __________________________________

## 7. Monthly Income

☐ No income

☐ Up to 1 minimum wage

☐ 1 to 2 minimum wages

☐ 2 to 3 minimum wages

☐ More than 3 minimum wages

☐ Prefer not to answer

## 8. Housing Situation

☐ Lives alone

☐ Lives with family

☐ Lives with partner

☐ Occupation / Informal housing

☐ Homeless

☐ Other: ___________________________________

## 9. Religion

☐ Catholic

☐ Evangelical

☐ Spiritist

☐ Umbanda/Candomblé

☐ Atheist

☐ Agnostic

☐ Other: ___________________________________

☐ Prefer not to answer

## 10. Relationship Status

☐ Single

☐ Dating

☐ Married / Civil union

☐ Separated

☐ Divorced

☐ Widowed

## 11. Age at First Sexual Intercourse (Coitarche)

Age at first sexual intercourse: ______ years

☐ Prefer not to answer

## 12. Number of Sexual Partners in the Last 12 Months

☐ None

☐ 1–5

☐ 6–10

☐ 11–15

☐ More than 15

☐ Prefer not to answer

## 13. Have You Ever Had Sex in Exchange for Money?

☐ Yes

☐ No

☐ Prefer not to answer

## 14. Health Insurance

☐ None

☐ Public system (SUS)

☐ Private health plan

Name of the plan (optional): ___________________________

____________________________	__________________________

Health professional’s signature	Date
