# Supplementary material for: Sociodemographic determinants of sexuality among transmasculine and transfeminine individuals: A cross‐sectional study in São Paulo, Brazil
Source: Int J Gynaecol Obstet. 2026 May 16;175(2):756–61. doi: 10.1002/ijgo.71081 (PMC13674826; doi:10.1002/ijgo.71081)
Supplement: Supplementary file 2 [file IJGO-175-756-s002.docx]

# Formulário Clínico Sociodemográfico – Ambulatório de Saúde Trans

## 1. Identificação

Nome social: ________________________________

Nome civil (opcional): ________________________

## 2. Identidade de Gênero

☐ Homem trans / Transmasculino

☐ Mulher trans / Transfeminina

☐ Não-binárie

☐ Outro: ____________________________

☐ Prefiro não responder

## 3. Cor/Raça (IBGE)

☐ Branca

☐ Preta

☐ Parda

☐ Amarela

☐ Indígena

☐ Prefiro não responder

## 4. Escolaridade

☐ Ensino fundamental incompleto

☐ Ensino fundamental completo

☐ Ensino médio incompleto

☐ Ensino médio completo

☐ Ensino superior incompleto

☐ Ensino superior completo

☐ Pós-graduação

☐ Prefiro não responder

## 5. Situação de Estudo

☐ Estudando atualmente

☐ Não está estudando

## 6. Situação de Trabalho

☐ Trabalho formal (carteira assinada)

☐ Trabalho informal

☐ Autônomo

☐ Desempregado

☐ Aposentado

☐ Estudante

☐ Outro: ____________________________

## 7. Renda Mensal

☐ Sem renda

☐ Até 1 salário mínimo

☐ 1 a 2 salários mínimos

☐ 2 a 3 salários mínimos

☐ Acima de 3 salários mínimos

☐ Prefiro não responder

## 8. Situação de Moradia

☐ Mora sozinho(a)

☐ Mora com família

☐ Mora com parceiro(a)

☐ Ocupação / Moradia informal

☐ Situação de rua

☐ Outro: ____________________________

## 9. Religião

☐ Católico(a)

☐ Evangélico(a)

☐ Espírita

☐ Umbanda/Candomblé

☐ Ateu(ateia)

☐ Agnóstico(a)

☐ Outro: ____________________________

☐ Prefiro não responder

## 10. Situação Conjugal

☐ Solteiro(a)

☐ Namorando

☐ Casado(a) / União estável

☐ Separado(a)

☐ Divorciado(a)

☐ Viúvo(a)

## 11. Início da Vida Sexual (Sexarca)

Idade da primeira relação sexual: ______ anos

☐ Prefiro não responder

## 12. Número de Parceiros(as) nos Últimos 12 Meses

☐ Nenhum

☐ 1–5

☐ 6–10

☐ 11–15

☐ Mais de 15

☐ Prefiro não responder

## 13. Já realizou sexo em troca de dinheiro?

☐ Sim

☐ Não

☐ Prefiro não responder

## 14. Plano de Saúde

☐ Não possui

☐ Sistema público (SUS)

☐ Plano/convênio privado

____________________________

Assinatura e Carimbo
